# *Candida albicans*-derived mannoproteins activate NF-κB in reporter cells expressing TLR4, MD2 and CD14

**DOI:** 10.1371/journal.pone.0189939

**Published:** 2017-12-27

**Authors:** Traci Ness, Mahmud Abdallah, Jaime Adams, Claudia Alvarado, Edwin Gunn, Brittany House, John Lamb, Jack Macguire, Emily Norris, Rebekah Robinson, Morgan Sapp, Jill Sharma, Ronald Garner

**Affiliations:** 1 Department of Biology, Armstrong State University, Savannah, Georgia, United States of America; 2 Department of Biomedical Sciences, Mercer University School of Medicine, Savannah, Georgia, United States of America; Louisiana State University, UNITED STATES

## Abstract

The ability of soluble *C*. *albicans* 20A (serotype A) mannoprotein (CMP) to serve as a ligand for toll-like receptor 4 (TLR4) and its co-receptors was examined using commercially available and stably-transfected HEK293 cells that express human TLR4, MD2 and CD14, but not MR. These TLR4 reporter cells also express an NF-κB-dependent, secreted embryonic alkaline phosphatase (SEAP) reporter gene. TLR4-reporter cells exhibited a dose-dependent SEAP response to both LPS and CMP, wherein peak activation was achieved after stimulation with 40–50 μg/mL of CMP. Incubation on polymyxin B resin had no effect on CMP’s ligand activity, but neutralized LPS-spiked controls. HEK293 Null cells lacking TLR4 and possessing the same SEAP reporter failed to respond to LPS or CMP, but produced SEAP when activated with TNFα. Reporter cell NF-κB responses were accompanied by transcription of *IL-8*, *TNFα*, and *COX-2* genes. Celecoxib inhibited LPS-, CMP-, and TNFα-dependent NF-κB responses; whereas, indomethacin had limited effect on LPS and CMP responses. SEAP production in response to *C*. *albicans* A9 *mnn4Δ* mutant CMP, lacking phosphomannosylations on *N*-linked glycans, was significantly greater (p ≤ 0.005) than SEAP responses to CMP derived from parental A9 (both serotype B). These data confirm that engineered human cells expressing TLR4, MD2 and CD14 can respond to CMP with NF-κB activation and the response can be influenced by variations in CMP-mannosylation. Future characterizations of CMPs from other sources and their application in this model may provide further insight into variations observed with TLR4 dependent innate immune responses targeting different *C*. *albicans* strains.

## Introduction

Pattern recognition receptors (PRRs) are expressed on a variety of cell types and are responsible for initiating immune responses to microbes and dead or dying cells [1–3]. Moreover, this action is achieved through PRR interactions with pathogen associated molecular patterns (PAMPs) and damage associated molecular patterns (DAMPs). The outcome of recognition often leads to an intracellular NF-κB signal that, in turn, promotes selected cytokine signals from innate immune cells. While responses to DAMPs and PAMPs are accepted as collateral occurrences during phagocytosis, the roles of non-phagocytic cells expressing PRRs have been frequently noted [4–6] but generally these remain underappreciated.

PRRs are of paramount importance in controlling and responding to indigenous microbiota, but the scope of their contribution is still being learned [7]. For example, there is minimal understanding of how the host might discriminate between noninvasive and invasive commensal threats. *Candida albicans* is a commensal yeast found on human mucosa and is recognized as a normal component of the gastrointestinal microbiota [8]. When the mucosal barrier is immunologically or physiologically compromised, this fungus can become an invasive pathogen. The clinical presentation of *C*. *albicans* infection on mucosal membranes is typically observed as an inflammatory disease of the mucosa that impacts the health and well-being of people worldwide [9]. In hospitals, disseminated candidiasis can emerge as life threatening fungal sepsis, particularly among neutropenic and non-neutropenic intensive care patients [10]. Invasive candidiasis, including both candidemia and deep-seated tissue candidiasis, occurs worldwide and the mortality rate has remained at approximately 40% [11]. In the ICU environment, the frequency of colonization may approach 80%, but recent surveys demonstrate that only 10% develop candidiasis [12]. Therefore, it is important that we examine all plausible mechanisms that might explain this confined virulence and/or susceptibility, viz., TLR4 recognition of *C*. *albicans*.

The most abundant *Candida* PAMPs on the cell wall surface are mannosylated polypeptides and polysaccharides (collectively referred to in this document as *C*. *albicans* mannoprotein or CMP). Moreover, host responses to CMPs follow a course of ligand detection, signaling, gene activation and cytokine production [13–16]. Phagocyte response to CMPs on the intact cell wall are known to utilize toll-like receptor 4 (TLR4), its co-receptors (MD2 and CD14) and mannose receptor (MR; CD206) recognition [13, 17]. In contrast to TLRs, MR is a membranous C-type lectin that lacks signaling motifs [18]. Nonetheless, MR is known to play an important role in the phagocytic process and is believed to cooperate with other PRRs in recognition [19, 20]. Confirming CMP recognition in cellular TLR4 models engineered to function independent of MR expression can further define CMP’s scope and variability as an inflammatory PAMP [21]. To this end, it is fortuitous that numerous PRR reporter cell lines have been engineered [22] to study potential PAMPs. Moreover, their commercialization has made this approach broadly available, reproducible and standardized [23].

With respect to CMP, recognition appears to be dependent upon the structural signature of the appended glycosylations. For example, Ueno et al. [24] reported that the loss of β-1,2-mannose residues from CMPs increased their inflammatory potential. NMR spectroscopy, combined with computational modeling, has revealed that internal mannosylation residues are immunodominant epitopes, but their recognition is also dependent upon the nature of the mannose unit at the reducing end of the di- and tri-saccharides [25]. Furthermore, differential expression of the *O*- and *N*-linked glycosylations of CMPs through genetic manipulation of *C*. *albicans* produces variations in host cell interactions and virulence [26, 27]. This dual recognition mechanism was further corroborated using PRR knockout mice wherein *O*- and *N*-linked glycosylation deficiencies affected PRR interactions with TLR4 and MR, independently [17].

Having previously reported that similar CMP preparations caused an increase in serum TNFα when injected into mice and induced murine macrophage production of TNFα *in vitro* (14, 15), our aim here was to examine TLR4 recognition of CMP without the participation of MR. Recent studies suggest that *in vivo* defenses to *Candida* challenge rely more on MR than TLR4 recognition [28], which correlates with our previous studies on hepatic *Candida* capture [29–31]. The studies by Netea et al. [28] also propose that TLR4 recognition of different *Candida* strains is dependent on variations in mannosylation patterns. To clarify and extend this concept, we began by examining *C*. *albicans* (20A; serotype A). CMPs from this strain were previously evaluated in multiple murine candidiasis studies [32–35]. Therein, the CMPs derived from strain 20A have been examined as both immune modulators and immunogens. To evaluate CMP here, we selected the engineered TLR4 model HEK-Blue h TLR4 cell line (HEK-TLR4; Invivogen, San Diego, CA) and its TLR4-negative HEK parental cell line (HEK-Null) because of their overall consistency, availability, controlled TLR4 expression and predictable ligand responsiveness. The correlation between TLR4, NF-κB and SEAP in this reporter cell line has been described in detail in the literature [36–38]. HEK-Null cells lack TLR4, MD2 and CD-14, but contain the same NF-κB SEAP reporter as HEK-TLR4 cells and were used as a negative control throughout the study. Our CMPs were extracted from *C*. *albicans* 20A, as previously described [33, 34, 39]. Potential endotoxin contamination was reduced by using baked glassware and endotoxin-free reagents. Any residual contamination was averted by depletion with polymyxin B resin. In addition to strain 20A, CMPs from *C*. *albicans* A9 (serotype B) and an A9 *mnn4Δ* mutant were used to examine TLR4 glycan discrimination in the absence of MR. Moreover, these findings corroborate the concept that strain variation can impact TLR4 recognition of CMP [28]. In general, this study demonstrates the utility of engineered TLR4-cell line models for assessing CMPs from different sources and evaluating quantitative or qualitative variations in glycan expression may impact immune recognition.

## Results

### CMP characterization

**Fig 1:**
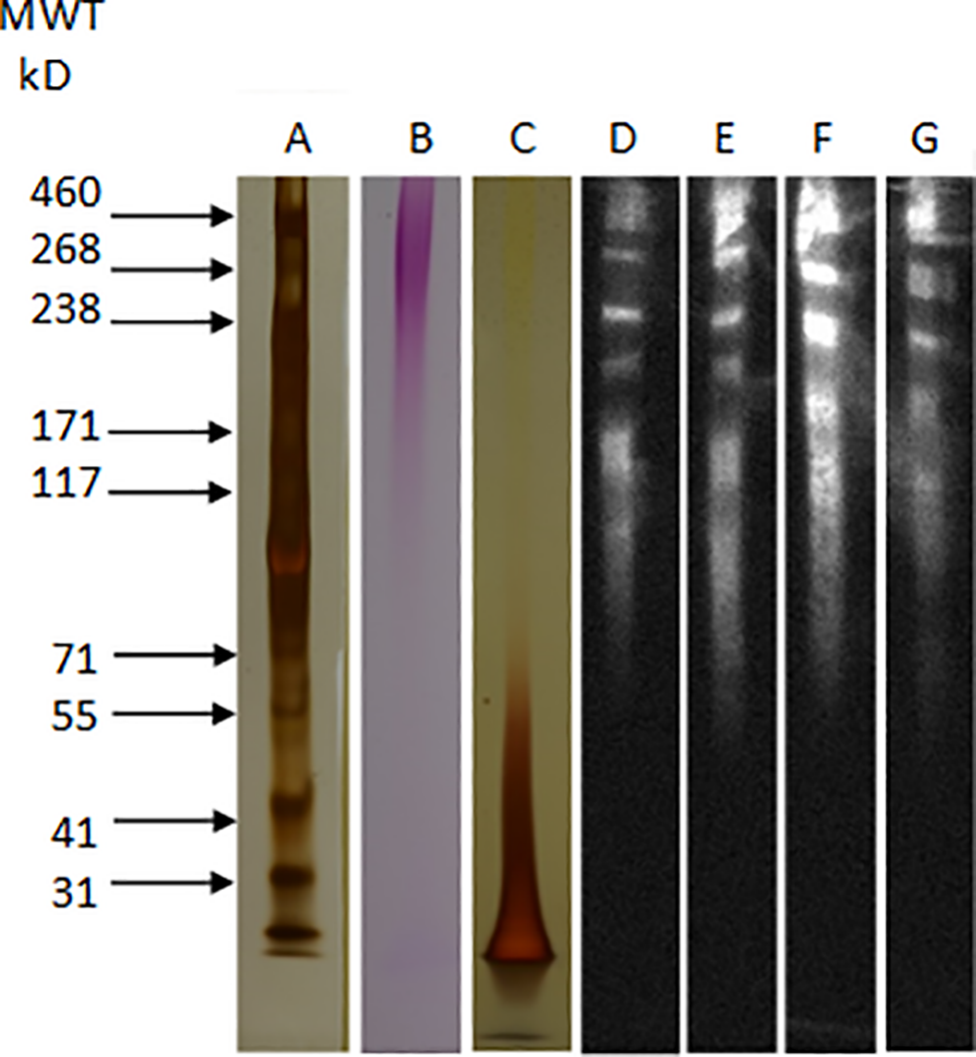
PAGE and Western blot analysis of CMP preparations. CMPs were combined with SDS sample buffer containing DTT to yield 1 mg/mL samples: 10ul of each sample was applied per well in 2–8% Tris acetate polyacrylamide gels and electrophoresed with a Tris/Tricine SDS running buffer. Western blot was performed in a standard discontinuous Tris/CAPS buffer. Gel lanes are: Lane A, molecular weight markers; Lane B, polysaccharide-stained CMP; Lane C, silver-stained CMP. Duplicate gels were also blotted to nitrocellulose. Lane D & E, 2 independent CMP samples probed with polyclonal rabbit anti-mannan antibody (Dako); Lane F & G, repeated 2 independent CMP samples probed with polyclonal rabbit anti-*C*. *albicans* antibody (Thermo Fisher). Chemiluminescent visualization of primary antibody binding used a goat anti-rabbit-peroxidase conjugate and a Super Signal West Pico Substrate Kit. These images are representative of 2 independent preps, wherein D and F represent a single CMP sample that displayed more activity than the E and G sample.

The CMPs prepared for this study contained approximately 8.5 mg/mL polysaccharide (measured by phenol sulfuric assay) and 0.46 mg/mL protein (determined by UV 230 nm/280 nm), representing a carbohydrate to protein ratio of 18:1. CMPs (7.5 μg/lane) were subjected to electrophoresis through 2–8% gradient polyacrylamide gels (Fig 1, Lanes A-E). Pierce HiMark (ThermoFisher, Waltham MA) pre-stained high molecular weight protein standards (Fig 1, Lane A) were used to estimate the mobility of CMP extraction products. Duplicate gels were stained for both polysaccharides (Fig 1 Lane B) and proteins (Fig 1, Lane C). Silver staining (Lane C) revealed a visible protein group in the CMP preparation that migrated as a smear from <25 kD through 70 kD, with a single distinct leading band of small proteins that migrated as 25 kD moieties. Polysaccharide staining of the gels revealed that the majority of the mannosylated CMP content traveled as expected for proteins with molecular weights ≥150 kD (Fig 1, Lane B). Duplicate CMP preparations from strain 20A displayed similar composition and were also active agonists for the HEK-TLR4 cells used in this study. A review of PAGE results found in other CMP studies suggests that the high molecular weight products in our preparations are equivalent to high molecular mass mannoproteins (HMM; >205kD) reported by Granger et al. [40]. These DTT-extracted CMPs demonstrated a slow trailing migration during electrophoresis, similar to what we observed with our own CMP moieties. This pattern may result from variable glycosylation produced during cell growth or the extraction process (i.e., acid lability or β-elimination) wherein a continuum of similar HMMs are produced. Upon treatment of HMM with PNGase F, Granger et al. [40] noted conversion of these to a non-glycosylated product that migrated and accumulated as a low molecular mass product, most likely the core proteins remaining after de-glycosylation.

Western Blot analyses using commercial rabbit anti-mannan antibody (Dako) revealed the presence of distinct mannan epitope moieties which migrated above 100 kD (Fig 1, Lanes D and E). Therein, polyclonal anti-mannan antibodies detected distinct bands at approximately 100, 170 and 225 kD. Above 225 kD, 4–5 bands were noted, one at approximately 250 kD and 3–4 clustered from 268 to 500 kD. The broad band noted at 170 kD may, in fact, consist of a series of 3 bands, but the resolution for the individual bands was inconsistent. The faster migrating band seen at 70 kD was preceded by a collection of antibody-reactive material from 65–100 kD. Immunoreactivity was not obvious below 65kD, which is near the initial point of silver reactive proteins. To compare with the Dako antisera findings, we also used anti-*Candida* antibodies (Thermo Fisher), which were obtained from rabbits immunized with whole *C*. *albicans*. These antibodies highlighted a banding pattern similar to that seen with anti-mannan antibodies (Fig 1, Lanes F and G), but with subtle differences in the bands identified below 260 kD. Therein, a different series of antigens were detected. For example, the 250 kD band migrated as a doublet in lane G but appears to be a single band in lane F. The 225 kD moiety migrated as a doublet in lane F, but as a singlet band in lane G. This may represent a distinct variation in glycan content or proteolysis that otherwise impacts a single glycoprotein. Although mannan- and *Candida*-specific antibodies demonstrated different results in the Western blots, both showed specificity for a similar family of large molecular weight moieties. Neither antibody bound to the low molecular weight products < 150 kD. In combination with the results from polysaccharide staining, these data suggest that the low molecular weight proteins are likely minimally glycosylated.

### HEK-TLR4 cells respond to CMP stimulation

**Fig 2:**
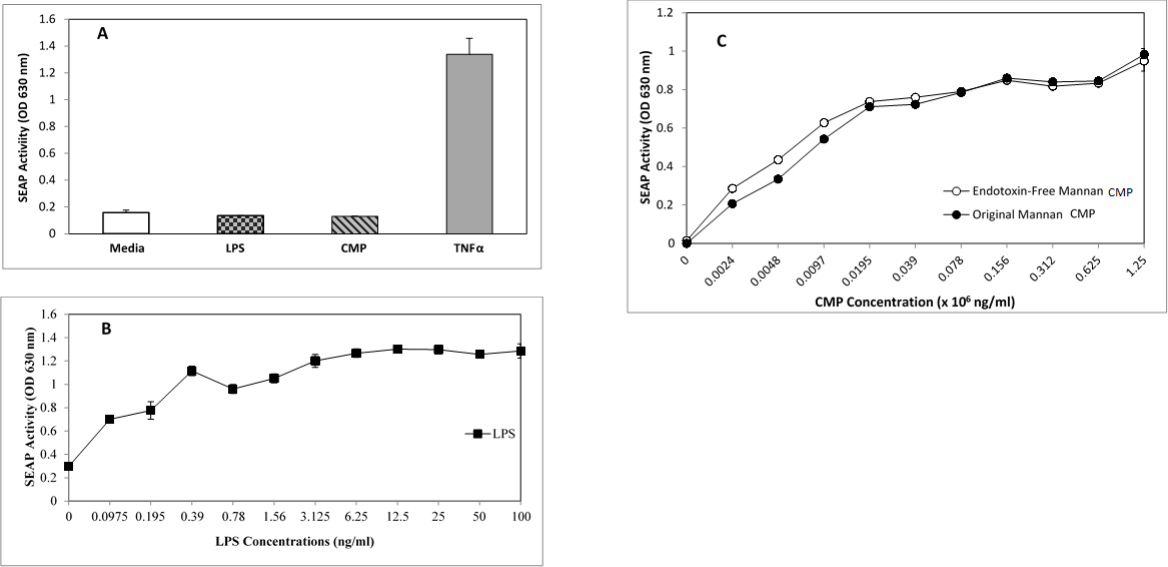
CMP activates TLR4 reporter cells in a dose-dependent manner. HEK-Null reporter cells lacking TLR4 (A) were unresponsive to media (clear bar) or stimulation with TLR4 ligands (LPS, checked bar and CMP, striped bar); however, TNFα treatment (positive control, gray bar) significantly activated NF-κB-dependent SEAP responses in the HEK-Null reporter cells. HEK-TLR4 reporter cells treated overnight with a range of LPS (B) concentrations reached a plateau of SEAP activity at 6.25 ng/ml LPS (closed squares). CMP stimulation of TLR4 reporter cells (C) was dose dependent and was not sensitive to LPS-depletion with polymyxin B; LPS-depleted CMP (open circles) or non-depleted CMP (closed circles). Control wells (designated by 0 on the X axis) were treated with complete media in the absence of TLR4 ligands.

**Fig 3:**
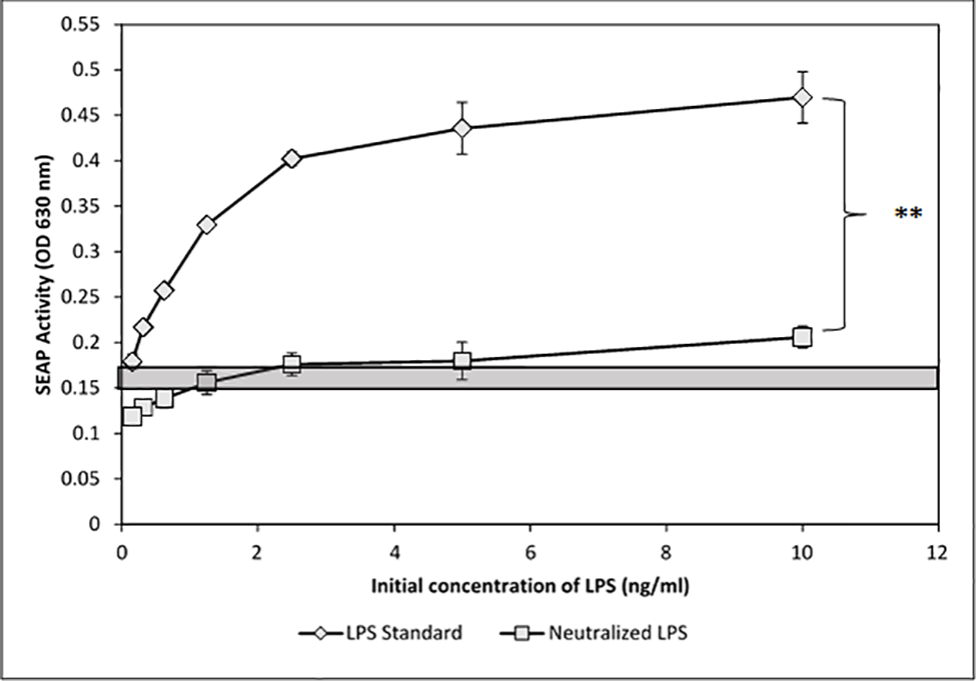
Polymyxin B depletion of LPS from spiked-positive control samples abolishes SEAP responses. To demonstrate the effectiveness of LPS depletion, samples of LPS-spiked media (0.16–10 ng/ml) were incubated twice on polymyxin columns with gentle rocking at room temperature. After elution from the columns, the effluents were applied to TLR4-reporter cells (as performed in Fig 2) and the stimulation was assessed with QuantiBlue. The gray bar represents the mean background of SEAP activity +/- SEM). The % net change in O.D. (HEK treatment O.D. minus background O.D.) produced by polymyxin depyrogenation of 10 ng/ml LPS was determined to be 89%. Significance is represented by the asterisks, p ≤ 0.005.

As expected, neither LPS nor CMP activated HEK-Null cells, as these cells lack TLR4 expression; however, a strong, TLR4-independent SEAP response was measured following TNFα treatment, facilitating the use of these cells as a negative control cell line for analysis of TLR4-specific activation events (Fig 2A). In contrast, LPS was a potent activator of HEK-TLR4 cells, producing measurable SEAP activity in response to concentrations > 0.10 ng/mL with maximal activation observed at ≥ 6.25 ng/mL LPS (Fig 2B). A similar dose-response curve was observed following CMP treatment but higher concentrations of this ligand were required to reach similar levels of SEAP activation (Fig 2C, filled circles). SEAP activity was detected following stimulation with ≥ 2.4 μg/mL CMP and the resultant activity increased in a dose-dependent manner, where activity plateaued following treatments with ≥156 μg/mL CMP. The maximum activations achieved for each of the agonists was roughly equivalent, but required broadly different amounts of each agonist (6.25 ng/mL LPS and 156 μg/mL CMP, respectively). Since LPS is such a potent TLR4 agonist and is found ubiquitously in the environment, it was important to demonstrate that the activity attributed to CMP was not the result of LPS contamination in the CMP preparation. Polymyxin B-resins are widely-used to remove LPS from a variety of sample types. When CMPs were incubated on polymyxin B columns, the dose response curves for SEAP activation among HEK-TLR4 cells were indistinguishable from those observed with the unfiltered CMP (Fig 2C). These results suggest that SEAP activation following CMP treatment is the result of CMP agonist activity and not due to LPS contamination. To confirm the LPS-neutralizing abilities of the column, we diluted LPS into media to achieve LPS concentrations ranging from 0.16 to 10 ng/ml. The dilutions were applied to the reporter cells as noted in Fig 2B and 2C. In this TLR reporter system, concentrations of LPS ≥ 6.25 ng/mL reproducibly yielded maximal SEAP activity and 0.16 ng/ml of our LPS standard was nearly undetectable by the reporter cells. After incubation on polymyxin B columns, depyrogenated and untreated LPS samples were applied to the reporter cells (Fig 3). The LPS concentrations shown in the Fig 3 legend are the LPS dilutions prepared before polymyxin treatments. Two 1 h incubations on the column reduced the ability of the LPS-spiked samples to activate the TLR4 pathway by 90–100%, while 3 1 h incubations produced a relative 100% reduction in activity. These data show that this method of depyrogenation can effectively remove 9–10 ng LPS (viz., roughly 100 endotoxin units; EU) from a 1 ml sample.

### Transcription of *IL-8*, *TNFα* and *COX-2* genes

**Fig 4:**
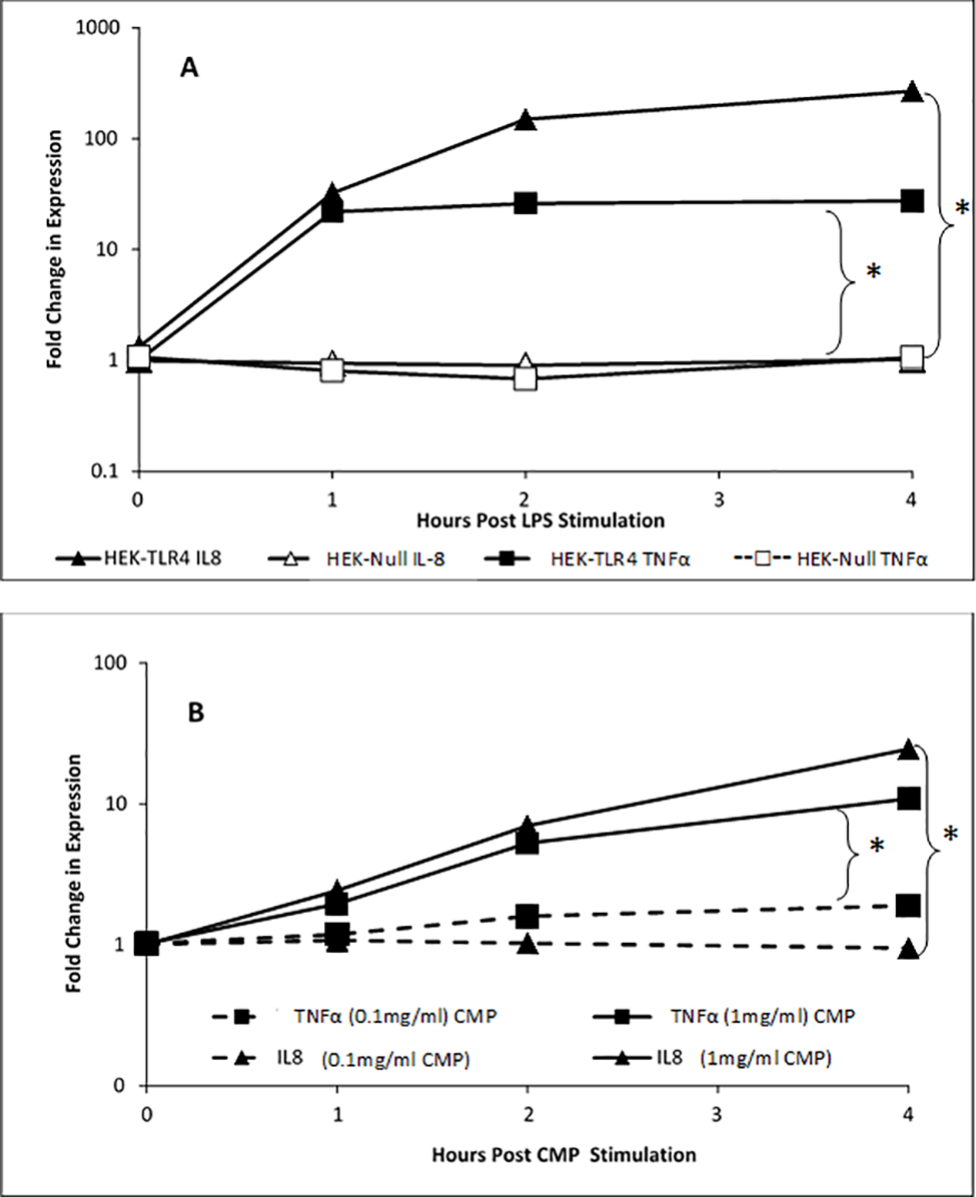
CMP-induced TLR4-signaling activates *TNF*α and *IL-8* mRNA expression. *TNFα* (squares) and *IL-8* (triangles) transcript levels were assessed by quantitative PCR. (A) HEK-TLR4 reporter cells (filled symbols) and HEK-Null reporter cells (open symbols) cells were stimulated with 12.5 ng/mL LPS for the indicated times prior to RNA isolation and qPCR analyses. (B) HEK-TLR4 reporter cells were treated with different doses of CMP (1 mg/mL, solid line; 100 μg/mL, dashed line) for the indicated times (0–4 h). Significance is represented by the asterisk, p ≤ 0.05.

**Fig 5:**
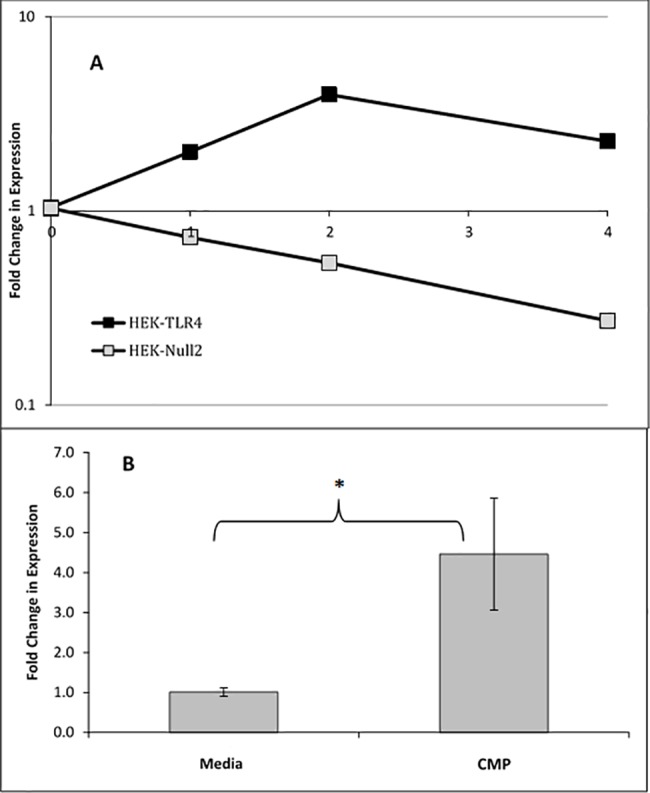
LPS- and CMP-mediated activation of TLR4 signaling induces *COX-2* expression. (A) HEK-TLR4 reporter cells (filled squares) and HEK-Null reporter cells (gray squares) were stimulated with 12.5 ng/mL LPS over 4 hours. Additionally, HEK-TLR4 cells were stimulated with 1 mg/mL CMP for 4 hours (B). Total RNA was isolated and subjected to quantitative PCR analysis. *COX-2* expression in individual samples was normalized to expression of *β-actin* and overall expression is relative to cells treated with media alone. Significance is represented by the asterisk, p ≤ 0.05.

Following TLR4-induced NF-κB-activation, transcription of pro-inflammatory cytokines and enzymes are viewed as the hallmark signature of TLR4 stimulation. To further elucidate the actions of CMPs as TLR4 ligands in the HEK-TLR4 model we measured inflammatory gene transcripts that are typically produced in phagocytic cell lines stimulated with *C*. *albicans* [41] or mannose-based PAMPs [42]. Given the manufacturer’s recommended assay time course and reduced cell density requirements (0.5–1 x 10^5^ cells/ml) for the reporter cell assays, initial cytokine ELISAs were unsuccessful. Therefore, we opted to examine mRNA transcripts for *TNFα* and *IL-8* genes 1–4 h after stimulation. Transcripts were detected within 1 h of stimulation with LPS or CMP. Both transcripts were significantly elevated (with *IL-8* expressed at the higher level) in HEK-TLR4 cells and the relative number of mRNA transcripts peaked for both genes between 1 and 2 h post-treatment (Fig 4A). RT-PCR analyses demonstrated that CMP also induced *TNFα* and *IL-8* transcription in a dose-dependent manner and that this response increased throughout the first 4 h of CMP treatment (Fig 4B). Furthermore, *TNFα* or *IL-8* transcripts were undetectable in LPS-treated TLR4-negative HEK-Null cells. Cyclooxygenase-2 (COX-2) is an enzyme that promotes prostaglandin synthesis and, as with TNFα and IL-8, can be induced following NF-κB activation. In this study, *COX-2* gene expression was induced within 4 h of LPS (Fig 5A) and CMP (Fig 5B) stimulation of HEK-TLR4 cells. No *COX-2* gene expression was detected when HEK-Null cells were treated with LPS (Fig 5A) or CMP (data not shown).

### Modulating CMP and LPS activation of NF-κB

**Fig 6:**
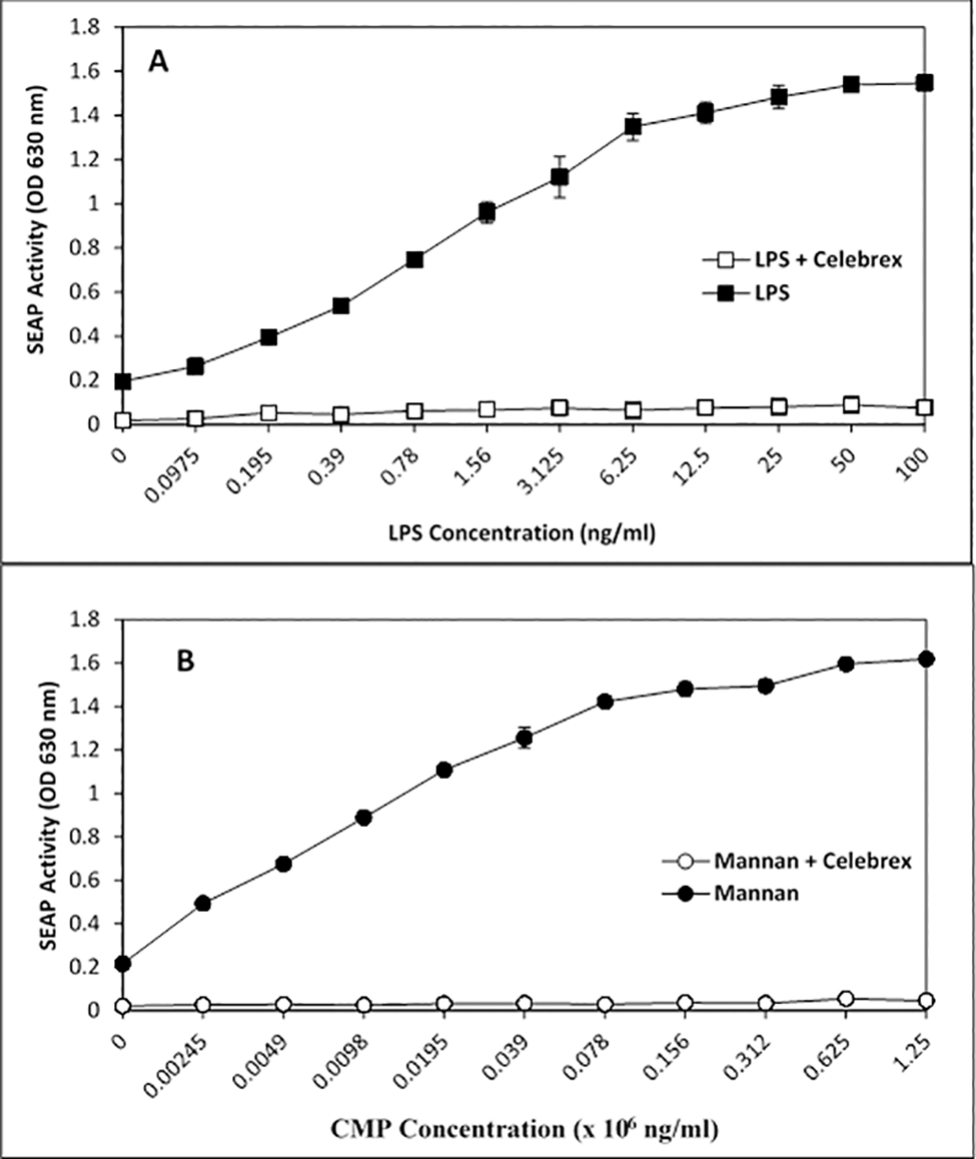
Celecoxib inhibits TLR4 activation. HEK-TLR4 cells were treated with media (filled symbols) or 100 μM celecoxib (Celebrex; open symbols) prior to LPS (A) or CMP (B) stimulation. SEAP activity was measured after overnight incubation.

**Fig 7:**
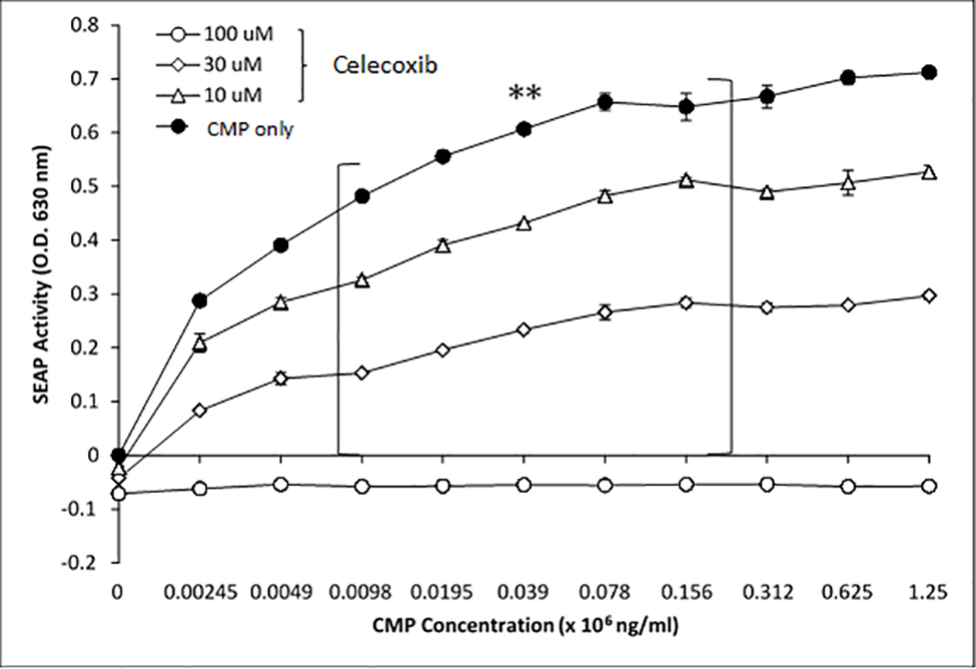
Celecoxib inhibits CMP activation of TLR4 in a dose-dependent manner. HEK-TLR4 cells were pre-treated with with 0 μM (filled circles), 10 μM (open triangles), 30 μM (open diamonds), or 100 μM (open circles) celecoxib for one hour prior to treatment with increasing concentrations of CMP as indicated. SEAP activity was measured the next day to evaluate cellular activation. CMP-induced SEAP activity was significantly reduced by celecoxib treatment (open symbols). Significance between the collective regressions is represented by the asterisks p ≤ 0.005.

**Fig 8:**
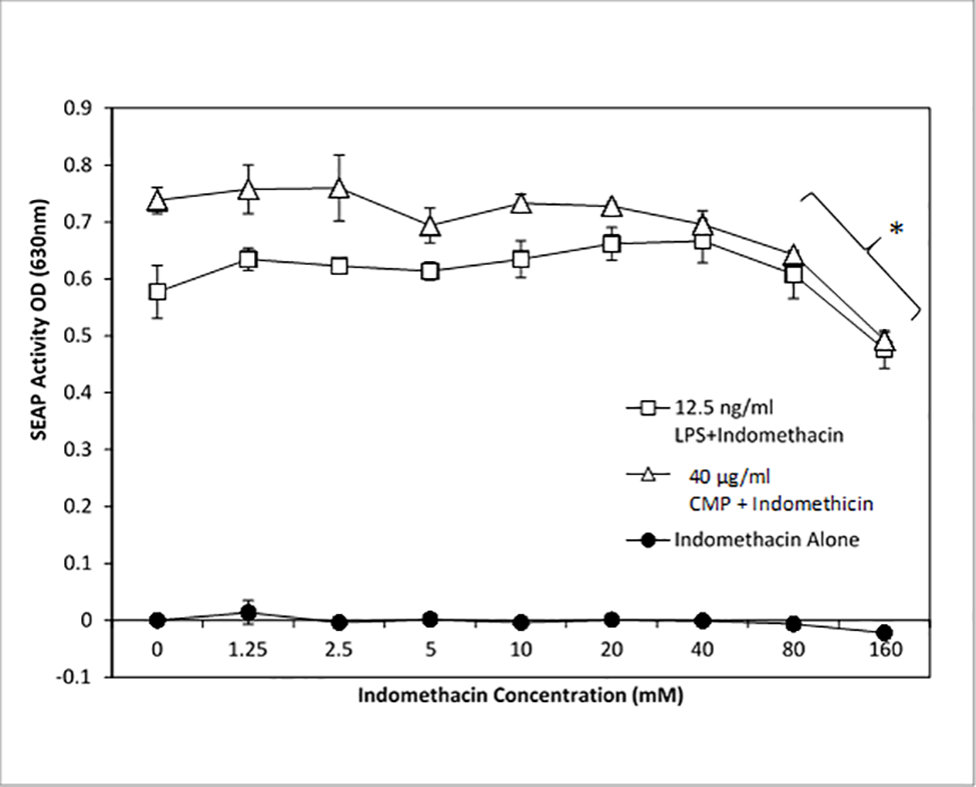
Indomethacin inhibits TLR4 activation only at high concentrations. HEK-TLR4 cells were pre-treated with a range of indomethacin concentrations (0–160 μM) for 1 h. After pre-treatment, cells were subjected to stimulation with media (filled circles), 12.5 ng/mL LPS (open squares) or 40 μg/mL CMP (open triangles). Ligand concentrations were selected after determining the lowest concentration required to achieve near-maximal SEAP activity. SEAP activity was measured after overnight incubation to evaluate cellular activation. The asterisk represents the significance (p ≤ 0.05) wherein the variance in mean SEAP activity of media plus indomethacin is compared with CMP or LPS treatments in the presence of 160 μM indomethacin.

**Fig 9:**
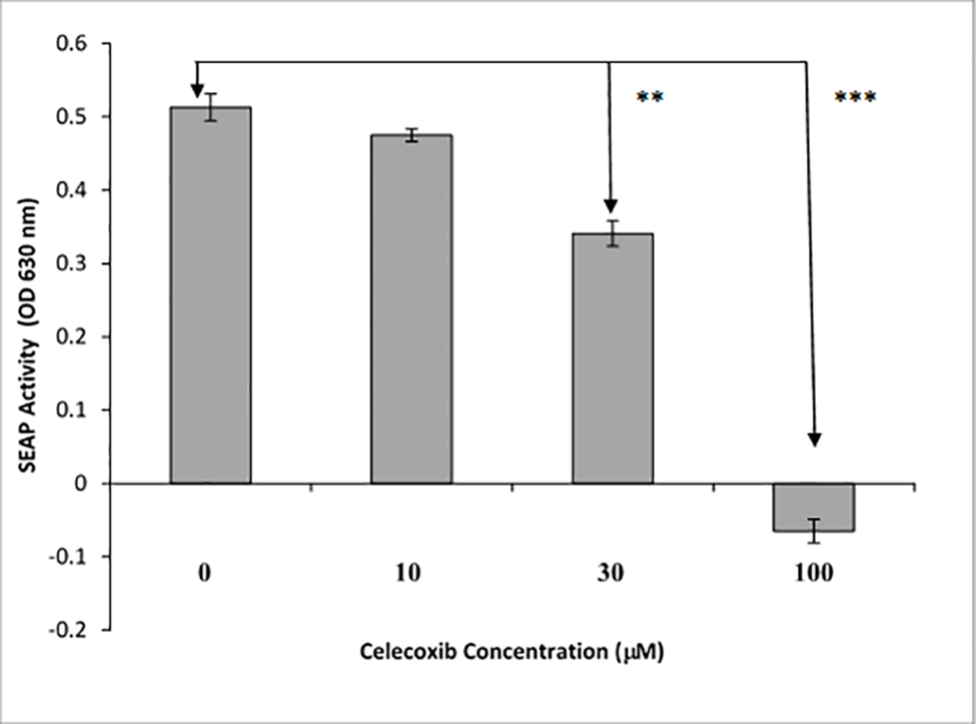
Celecoxib inhibits TNFα-induced activation of HEK-TLR4 cells in a dose-dependent manner. HEK-TLR4 cells were treated with varying concentrations of celecoxib for one hour after which cells were stimulated with TNFα (2μg/well). After overnight incubation, SEAP activity was measured to evaluate cellular activation. Significance is represented by the asterisks, p ≤ 0.0005.

**Fig 10:**
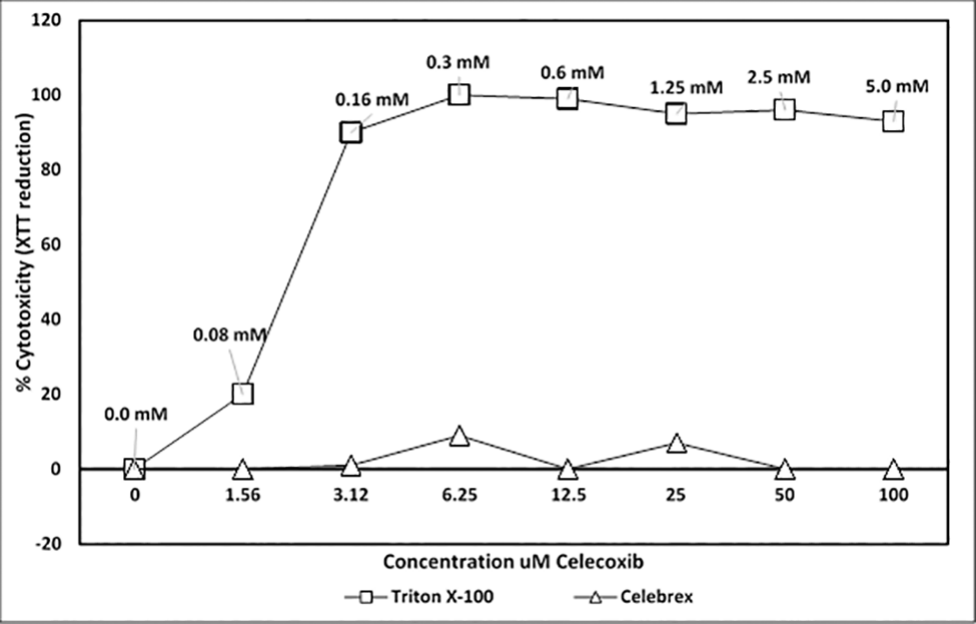
Celecoxib-treatments below 100mM are not cytotoxic to HEK-TLR4 cells. The HEK reporter cells were cultured overnight in dilutions of celecoxib (triangles) or Triton X-100 (control; squares). The X axis shows the final concentrations of celecoxib in the culture media and the numerical labels above the square data labels show the mM concentrations of Triton X-100 used in the positive cytotoxic controls. Cell viability was tested using XTT + menadione. Substrate (XTT) conversion was monitored and measured as OD 490 during a 12 h incubation. Net absorbance was adjusted by subtraction of media only (acellular) absorbance and % cytotoxicity was computed as an absorbance quotient using treated cell XTT conversion/non treated cell XTT conversion x 100%. Computed SEM were negligible and while shown in the graph, they are hidden by the symbols.

To further correlate CMP treatment with NF-κB activation, we utilized celecoxib to inhibit NF-κB signaling. Celecoxib (active agent in Celebrex), a selective pharmaceutical inhibitor of COX-2, prevented LPS- and CMP-induced NF-κB activation in HEK-TLR4 cells (Fig 6). The inhibition was dose-dependent insomuch as 100 μM celecoxib treatment completely abrogated the CMP response (p ≤ 0.005), while 10 μM and 30 μM treatments with celecoxib demonstrated intermediate levels of inhibition (Fig 7). In contrast, indomethacin (another COX inhibitor) had very little effect on NF-κB activation irrespective of the agonist (Fig 8). To better demonstrate inhibition, optimum concentrations (lowest concentration required to achieve near-maximal SEAP activation) of LPS and CMP (12.5 ng/mL and 40 μg/mL, respectively) were selected and applied to reporter cells. No effect was seen when HEK-TLR4 cells were pre-treated for 1 h with 1.25–80 μM indomethacin prior to the addition of LPS or CMP. However, incubation with 160 μM indomethacin resulted in approximately 25% reduction (p ≤ 0.05) of the LPS and CMP-induced SEAP responses. TNFα initiates a potent SEAP response in both HEK-TLR4 (Fig 9) and HEK-Null cells (Fig 2A). This response was significantly inhibited (p ≤ 0.005) in a dose-dependent manner when cells were pre-treated with celecoxib prior to TNFα stimulation. Complete ablation of the response was observed with 100 μM celecoxib pre-treatment. These findings directly paralleled the dose-dependent celecoxib kinetics observed with CMP-dependent NF-κB activation (Fig 8). Cytotoxicity assays using metabolic XTT reduction were performed to evaluate any changes in HEK-TLR4 reporter cell viability that might be attributable to the addition of celecoxib (Fig 10). Cells were incubated overnight with the same dilutions of celecoxib used to modulate NF-κB-dependent SEAP production. No cytotoxicity was attributable to celecoxib treatments. In contrast, triton X-100 dilutions (16 mM or greater) produced > 85% cytotoxicity which mirrors values reported by other studies [43].

### Role of *N*-linked glycosylations in TLR4 recognition

**Fig 11:**
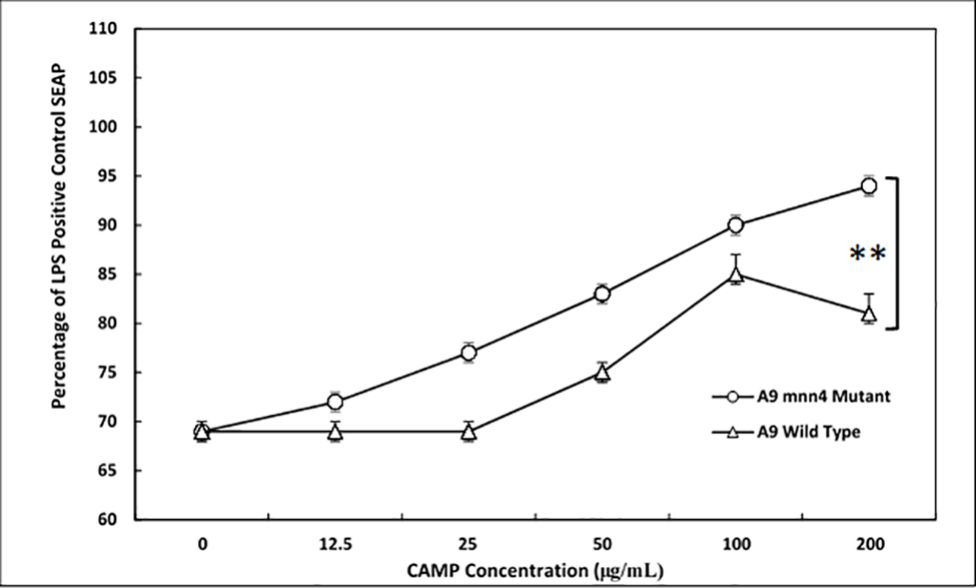
CMPs derived from *C*. *albicans* A9 and A9 *mnn*4*Δ* mutant strains both stimulate HEK-TLR4 cells. The A9 *mnn*4 *Δ* mutant CMP lacks phosphodiester-linked extensions on the *N*-linked glycans and was found to be superior to CMP from the A9 parent strain as a TLR4 agonist. In this experiment SEAP conversion was stopped at 45 min. Peak LPS stimulation corresponded to mean absorbance of 0.11 +/- 0.004 absorbance units (positive control). The negative control wells received only additional media. All data were converted to percentage of LPS control by dividing the mean net absorbance of CMP treated cells by the mean net absorbance of LPS treated cells. SEM was converted to % SEM in the same manner. Significance that is represented by the asterisks was p ≤ 0.005.

*Candida* strains possessing knockout mutations in the MNN4 gene have been used to determine the contributions of *N*-linked glycosylations to cellular hydrophobicity [44]. The changes in glycan charge that accompany loss of MNN4 are attributable to loss of the phosphoglycan side chain and correlate with a failure to bind to Alcian Blue. This change includes the loss of β-1,2-oligomannosides that are linked to N-linked glycan through mannosylphosphate [45]. To correlate changes in CMP composition with TLR4 recognition we compared CMPs derived from *C*. *albicans* strains A9 and A9 *mmn4*Δ (serotype B strains) [44] in TLR4 reporter cells. The data are shown in Fig 11 as percentage of the paired LPS-induced SEAP controls. SEMs are shown, but their incrementally low values placed them within the domain of the symbols. CMP from both the A9 parent strain and the A9 *mnn4Δ* mutant induced NF-κB SEAP production, but A9 *mnn4Δ* mutant strain-derived CMP was significantly (p ≤ 0.005) more potent than the parent A9-derived CMP (Fig 11). This may be interpreted to suggest that the β-1,2 mannan chains normally associated with phosphoglycan extensions on *N*-linked glycosylations could possibly interfere with interactions between TLR4/co-receptor complex and CMP. For example, these glycans may mask shorter *O*-linked serine glycosylations, compete for binding sites or change the hydrophobic/hydrophilic nature of CMPs. Moreover, this alteration of molecular charge may allow A9 *mnn4Δ* mutant CMP better interaction with TLR4 and/or its co-receptors thereby resulting in a more competent activation platform on the membrane.

## Discussion

We previously reported that CMPs stimulate macrophages *in vitro*, causing these cells to produce TNFα [14]. It has since been demonstrated that TNFα production in response to CMP requires the sequential actions of TLR4, MyD88, and NF-κB [13]. Since TLR4 cooperates with CD14 and MD2 in ligand recognition, we used a reporter cell line that expresses an engineered and functional TLR4 receptor complex and a NF-κB-dependent SEAP-reporter system (Invivogen). Many different cell types express TLR4; whereas, display of co-receptors CD14 and MD2 is primarily restricted to immune cells. However, structural cells have been shown to participate in ligand recognition via surface-expressed TLR4 and soluble co-receptors [46]. Thus, lingering questions regarding the role of MD2 and CD14 in TLR4 activation by non-LPS ligands require additional research. The primary goal of this study was to assess CMP as a TLR4 PAMP using an established reporter cell [22] that also lacked MR. CMPs derived from *C*. *albicans* 20A have been shown to stimulate TNFα production in mice (14) and cultures of murine macrophages (15). These and other CMPs from various preparations contain similar electrophoretic patterns that display a defined reactivity with anti-mannan antibodies. The overall aim of the study was to compare and contrast CMP induced TLR4 activation with LPS-induced activation in the same reporter cell system. In accomplishing this goal, the data show that CMP contains multiple high molecular weight moieties that could serve as molecular vehicles in the delivery of the ligand signals to TLR4/MD2/CD14. It was clearly shown that CMP can produce a reporter cell response equivalent to that observed with LPS, provided sufficient quantities of CMP are used. Using a depyrogenation system, it was conclusively demonstrated that the CMP-induced TLR4 responses were not due to endotoxin contamination. Despite the fact that this cell line contains an imported and engineered NF-κB-dependent reporter system, these data also showed that the stimulated reporter cells not only produced SEAP but also transcribed CMP-inducible mRNA transcripts from inflammatory cytokine genes (viz., *TNFα* and *IL-8*). These cytokines, as well as COX-2 are commonly observed in CMP responses among cultured phagocytes. In this respect, HEK 293 cells have also been shown to be capable of producing IL-8 in an NF-κB-dependent fashion when stimulated with TNFα [47]. COX-2’s role in the response was further examined using COX inhibitors. Celecoxib was used as a modulator of NF-κB-dependent cellular regulation, wherein it was found that COX-2 may function in an auto-regulatory mechanism that possibly sustains the initial reporter cell response to CMP [28]. Insomuch as human macrophages respond to mannose-containing PAMPs with strong prostaglandin E_2_ (PGE_2_) responses [48], contrasting this modulation against a COX-1 inhibitor provided some indirect assurance that the NF-κB inhibition was dependent on COX-2. Finally, the correlation of variable TLR4 reporter cell responses to differentially expressed β-1,2 mannosylations paralleled and confirmed the findings of other labs regarding pattern recognition of *C*. *albicans* cell wall glycans. In summary, these TLR4-reporter cells are a flexible genetic platform that are robust and easily transfected with plasmids containing variable collections of PRR genes. These features make them an appropriate cellular model for evaluating genetic variations in TLR4 and co-receptor dependencies that may contribute to variable host recognition of *C*. *albicans* strains. Moreover, PRR-dependent reporter cells also provide a valuable tool for examining variations in glycan expression on CMP that may contribute to virulence.

The biochemical basis of TLR4 reactivity with CMP is dependent upon glycosylation patterns. CMPs derived from *C*. *albicans* by citrate extraction and CTAB purification possess both *N*- and *O*-linked mannosylations [44, 49]. *O*-linked mannosylations are composed of 3–4 α-1,2-linked mannosides that are anchored to CMP at Ser/Thr residues. *N*-linked mannosylations are attached to CMP at Asn-*X*-Ser/Thr sequons and are much larger and more complicated polymers that contain a mixture of various β- and α-linked mannosides occurring as branching mannose polymers. PRR recognition and activation with these glycosylations may well be dependent upon glycan linkages and quite possibly dependent upon their presentation as a part of a larger multiplexed molecule. For example, synthetic forms of mannans have been compared with native CMPs for their ability to stimulate TNFα secretion in macrophage-like cells, but little if any stimulation was noted among the synthetic mannose polymers [50]. In vaccine studies, synthetic conjugates of mannans coupled to proteins provide protective immunity in mice [51]. Taken together, these results indicate that the native state and form of CMP maybe critical to recognition. Other studies [13, 52] have suggested that *O*-linked mannosylations on CMP react with TLR4 and activate the associated pathways leading to NF-κB release. Among wild type serotype A stains of *C*. *albicans*, β-1,2-mannosylations are expressed on acid-labile phosphoglycan extensions of the *N*-linked glycans and are also found on the termini of acid-stable extensions. Epitopes formed by the β-1,2-extensions appear to be the predominant target (viz., antigenic factor 5) in antisera raised against *C*. *albicans* mannans [53]. Furthermore, studies with dendritic cells have shown that protein-free β-1,2 mannosides reduce the production of inflammatory cytokines [24]. These researchers also found that the inhibitory activities of the β-1,2 mannosides were sensitive to polyclonal rabbit anti-mannan antibody neutralization. These studies collectively point to a regulatory action that is dependent upon expression of β-1,2-linked mannosides.

In the present study, the data showed that both LPS and CMP achieve similar maximal thresholds of NF-κB activity in TLR-4 reporter cells, but very different ligand concentrations were required for their respective maximal activations. Thresholds can result from extracellular or intracellular regulation, but may also depend on ligand presentation where competitive agonists and antagonists (e.g., β-1,2-linked mannosylations) interfere or enhance interactions with the receptor. It has also been proposed that TLR4 activation may be regulated by internal cellular signals (e.g., eicosanoids) that act through G-coupled protein kinase A anchoring proteins (AKAPs) [54]. This work suggested that LPS-induced PGE_2_ suppressed TLR4-induced TNFα production in macrophages through the actions of AKAP8-anchored protein kinase A-RII. However, they also showed that endogenous PGE_2_ induced cAMP which provided a positive AKAP10 signal for cytokines IL-6 and IL-10 that also enhanced nitric oxide production. The fact that our data showed both LPS and CMP induced transcription of COX-2 in the reporter cells and both NF-κB responses were inhibited by a COX-2 inhibitor supports the concept that endogenous PG may also play a positive role in controlling TLR4 responses in reporter cells. For example, it is well known that celecoxib directly inhibits COX-2 and decreases the production of PGs. This regulatory feature could have divergent effects on NF-κB related actions insomuch as PGE_2_ has been shown to promote the transcriptional activities of NF-κB, while cyclopentenone PG has been shown to inhibit IκB kinase [55]. These and other authors (46) propose models wherein PGs impact the early response to a TLR4 ligand by initially amplifying and sustaining NF-κB’s activity. Another study [56] showed that celecoxib depletion of intracellular PGE_2_ enhanced NF-κB p65, but inhibited p50 translocation to the nucleus and interfered with p50 binding to DNA. Thus, our application of celecoxib and indomethacin during the early stages of the reporter cell response to CMP and the subsequent inhibition of NF-κB allowed us to consider the assumption that the reporter cell NF-κB responses to either CMP and/or TNFα may partly depend upon endogenous PGE_2_ as a part of an early PG amplification signal.

Moreover, celecoxib was an effective inhibitor of both LPS and CMP stimulation in the HEK-TLR4 model. Complete inhibition of CMP was achieved with 100 μM and 50% inhibition was measured in the presence of 30 μM celecoxib. This agrees with other studies that report 50% inhibition of TNFα-induced activation when cancer cells are treated with 27 μM celecoxib [57]. In contrast, indomethacin in our model was found to be a weak inhibitor of LPS- and CMP-induced NF-κB activation, insomuch as >100 μM concentrations of the drug were required to achieve 25% inhibition. Similar to previous reports in other cell lines, we found celecoxib was superior to indomethacin in inhibiting NF-κB activity [57]. For example, Takada et al. (2004) reported that 600 μM indomethacin was needed in a tumor model to achieve 50% inhibition of TNFα-induced NF-κB release. After overnight exposure to higher concentrations of indomethacin, the HEK-TLR4 cells were rounded and less adherent, but otherwise appeared healthy and viable. Takada et al. previously reported that indomethacin concentrations above 100 μM could produce a 25% decrease in viability/proliferation following 2 days of exposure, as compared to our overnight exposure. It is well-recognized that indomethacin is predominantly a COX-1 inhibitor that will also inhibit COX-2, while celecoxib is a selective COX-2 inhibitor. However, we should not assume that the variation in the action of COX inhibitors in CMP or LPS activation was solely dependent upon PG production. Nonetheless, the celecoxib sensitivity we observed agrees with recent studies in which alternative NSAID-dependent control mechanisms have been proposed. For example, another plausible point for celecoxib-sensitivity is the NF-κB interference mechanism that has been identified to be dependent upon non-steroidal anti-inflammatory drug induced gene-1 (NAG-1) products [58]. Celecoxib interference with NF-κB activation in the current HEK-TLR4 model could also occur through NAG-1 modulation. In conclusion, similar reporter cell sensitivities to COX-1 and -2 inhibitors by LPS- and CMP-treatments suggest that their respective PG activation mechanisms are convergent with respect to NF-κB activation and modulation.

The observation that the TLR4-reporter cells were more sensitive to LPS than CMP suggests variable activation thresholds that may result from either variable ligand efficiencies or variable regulation. While HEK-TLR4 cells produced a maximal SEAP response to both ligands, LPS served as the more potent agonist. This observation was further evaluated by comparing the estimated ligand molarities used for each of the two ligand stimulations. We have determined that our CMPs are >200kD, whereas the *Salmonella* LPS used in this study is recognized to be only 50-100kD (Sigma Technical Bulletin; Product L2262). When the masses of each ligand are considered, the molar values required to induce 50% SEAP activity were 0.5–1.0 nM and 40 μM for LPS and CMP, respectively. Therefore, in regard to TLR4 stimulation, *Salmonella* LPS was estimated to be 40,000–80,000 times more potent than our CMPs. If maximal SEAP activation is used as an indirect measurement of receptor or signal saturation, we can surmise that saturation may occur with 125–250 nM LPS or 5 mM CMP. These values suggest that LPS is 20,000–40,000 times more active than CMP as a TLR4 agonist. Other studies support our findings in that 10–100 μM concentrations of *Candida* mannan are needed to induce TNFμ production by the macrophage cell line J774.2 [50]. The differences in the molar requirements for TLR4 activation by LPS and CMP may be the result of differences in ligand-binding affinities or receptor complex composition (e.g., co-receptors required). It may also be considered that the assembly and sorting of multiple co-receptors into their effective molecular ratios is favored by the LPS ligand.

When polymyxin B columns were used to remove potential endotoxin contamination from CMP preparations, no change was noted in SEAP responses. In contrast, media spiked with 0.16–10 ng/ml LPS lost 90–100% of its stimulatory activity when exposed to polymyxin B resin. The minimum threshold of LPS detection for these reporter cells was 0.32 ng/ml LPS. Based on our calculations, CMP produces a near maximum threshold SEAP response at 78 μg/ml, which should hypothetically contain no more than 10.4 ng/ml LPS. The polymyxin B columns effectively reduced the TLR4 stimulation induced by 10 ng/ml LPS by 90%, but no change was noted in polymyxin B treated CMP at 78 μg/ml. We can conclude from these data that the CMP responses observed here with TLR reporter cells are not the result of endotoxin contamination.

It is generally known that NF-κB activation occurs following the phosphorylation and release of its inhibitor IκB [1]. NF-κB travels to the nucleus where it cooperates with other transcription factors to bind to the promoters of specific genes. The NF-κB signal derived from TLR4 activation drives transcription of *TNFα* [59] and other cytokine genes [60]. CMP treatment of TLR4-reporter cells was followed by a 10-fold increase in *IL-8* and *TNFα*, like what has been reported after LPS exposure. CMP enhanced *COX-2* gene transcription 2-4-fold compared to cells treated with media alone. Taken together, these data show that CMP activates a similar cascade of signaling events to those seen following LPS binding to TLR4 and its co-receptors [60, 61]. This finding further supports the use of HEK-TLR4 cells as a representative TLR4-reporter cell for comparative recognition of mannans and mannoproteins from different *C*. *albicans* strains. Moreover, recognition was dependent upon ligand concentration, occurred independently of MR expression and produced detectable transcriptional products.

The data reported here indicate that CMPs induce NF-κB in HEK-TLR4 cells in a fashion similar to LPS. CMP activation is most likely dependent upon the shorter *O*-linked mannosylations present on a soluble, high molecular mass mannoprotein that is extracted with hot citrate from *C*. *albicans* 20A. TLR4 recognition of CMP can occur independent of MR, but the dependency of CMP recognition on CD14 and MD2 is still unknown. Nonetheless, it has been suggested that variations in β-1,2-linked mannosides occurring on the *N*-linked glycans influence cytokine production, a function that is otherwise currently attributed to *O*-linked recognition [24]. Furthermore, the contrasting TLR4-reporter cell responses observed with A9 and A9 *mnn4Δ* mutant CMPs support the notion that strain-dependent variation in *O*-linked phosphoglycan extensions of *N*-linked glycosylations may interfere with TLR4 recognition of *O*-linked mannosylations. This interference may be due to altered hydrophobicity and/or changes in the molecular arrangement of glycans wherein masking of TLR4 targets occurs. In the absence of MR, the TLR4/MD2/CD14 receptor complex is acting without the benefit of this *N*-linked glycan receptor, but even in its absence an NF-κB activation signal is produced in the reporter cell. The data presented here showed that loss of *MNN4*-dependent glycans on CMP enhanced the stimulatory nature of CMP extracted from the A9 *mnn4* Δ-mutant.

Taken together, the data in this study demonstrates that the HEK-TLR4 model is suitable for assessing the interactions of CMPs with TLR4 and its co-receptors. The response progresses much like responses seen with the human macrophage cell line, THP1. Moreover, the model provides insight into a possible mechanism that non-phagocyte TLR4 expression might provide to initiate an innate immune response in the absence of MR. The studies described here also showed that NF-κB responses by the TLR-4 reporter cell line reflected subtle changes in the expression of mannosylations on soluble CMP. The standardization of this model will provide a testing platform for genetic manipulation of PRR and the evaluation of *C*. *albicans* strain-dependent variations in TLR4 recognition.

## Materials and methods

### *C*. *albicans* cultures and CMP extraction

CMPs were extracted from *C*. *albicans* 20A (a serotype A, an isolate originally obtained from Errol Reiss, Centers for Disease Control, Atlanta, GA) using hot citrate buffer and cetyltrimethylammonium bromide (CTAB; also referred to as cetavalon) purification [39, 49]. A9 (serotype B) and A9 *mnn4Δ* mutant-derived CMPs were a gift from Kevin Hazen, Duke University [44]. All buffers were prepared with endotoxin-free water (EF; 0.001 EU/mL) derived from a Millipore Academic-Elix System with a BioPak Filter (EMD Millipore, Billerica, MA). All glassware was baked at 250°C for 2 h. *Candida* stocks were maintained at 4°C on Sabouraud dextrose agar (Difco Laboratories, Detroit MI). Prior to bulk culture, an overnight culture was prepared by inoculating 200 mL of Sabouraud dextrose broth and shaking (150 rpm) at 35°C. The starter culture was distributed to eight 4 L flasks containing 1 L Sabouraud dextrose broth with 250 μg/mL ampicillin (Sigma, St Louis, MO) and grown with shaking overnight at 35°C. Cells were pelleted by centrifugation at 2840 × g, washed with 500 mL sterile, EF-PBS (Sigma) and suspended in 200 mL of 0.02M citrate buffer (pH 7.0). The cell suspension was autoclaved at 125°C in the citrate buffer for 90 min. The autoclaved cells were pelleted at 2840 × g, suspended in a fresh 200 mL of citrate buffer and again autoclaved for 90 min. Both citrate supernatants were combined and either precipitated in an equal volume of methanol or lyophilized. The crude mannan preparation was dissolved in 200 mL water and dialyzed against 4 changes of EF-water during a 48 h period. The dialyzed sample was lyophilized, weighed, and suspended in EF-water at a concentration of 4 g/100 mL. The volumes described in the following steps were adjusted to be proportional to 4 g of crude mannan/100 mL water. Approximately 100 mL of the crude cell wall extract was combined with 4 g CTAB/150 mL of water, stirred and allowed to set at room temperature overnight. The precipitate was removed by centrifugation at 3500 × g, suspended and washed in 50 mL EF-water for 1 h. The CTAB precipitates were discarded. The supernatant (150 mL) was combined with the 50 mL wash and 100 mL of 1% (wt/vol) boric acid. The CMP suspension was adjusted to a pH of 8.8 by the slow addition of 1 N NaOH while stirring at room temperature for 1 h. The precipitate was collected by a 15 min centrifugation at 3500 × g and was washed with 100 mL 0.5% (wt/vol) sodium acetate (pH 8.8). The sodium acetate solution was eluted from the precipitate and discarded. The precipitate was dissolved in 50 mL of 2% (vol/vol) acetic acid. Next, 1 g of sodium acetate in 3 vols of ethanol was added to re-precipitate the crude mannan. The precipitate was collected by centrifugation at 3500 × g, dissolved in EF-water and the cloudy solution was clarified by adjusting the pH to 7.0 with the slow addition of 1N NaOH. The CMP solution was dialyzed against 4 changes of EF-water over 48 h. The solution was rapidly frozen using a methanol/dry ice bath and lyophilized. CMP was stored as a lyophilized powder at 4°C.

### SDS polyacrylamide gel electrophoresis and immunoblotting

CMPs were subjected to electrophoresis through 10 cm Tris-acetate gels prepared as 2–8% acrylamide gradients (Thermo Fisher Scientific Inc., Waltham, MA). The samples were prepared for electrophoresis by boiling for 2 min in sample buffer containing 20 mM Tris-HCl (pH 7), 2% SDS, 100 mM DTT, 10% glycerol and 0.02% bromophenol blue. Gels were placed into Tris-Tricine SDS buffer (Expedeon Running Buffer; Thermo Fisher) and samples were subjected to a constant current of 7.5 mA per gel for approximately 1 h or until the dye front progressed to the bottom of the gel. Pre-stained Precision Plus Protein Dual Xtra standards^TM^ (BioRad) or Pierce HiMark standards (Thermo Fisher) were included on each gel to assess electrophoretic mobility. Gels were either fixed and stained immediately or transferred onto nitrocellulose. Staining was performed according to manufacturer’s directions with a standard coomassie blue, silver or polysaccharide staining kit (all from Thermo Fisher). Transfer to nitrocellulose was performed with a semi-dry system using filter papers soaked in discontinuous anode and cathode buffers. The first anode filter was saturated with 0.3 M Tris at pH 10.4 in 30% (vol/vol) methanol. The second anode filter was saturated with 0.25 M Tris at pH 10.4 in 30% (vol/vol) methanol. The cathode filter was saturated with 0.025 M Tris/0.4 M amino caproic acid/0.00044% SDS at pH 9.4 in 30% (vol/vol) methanol. CMPs were transferred at 80 mA for 1 h onto NitroPure^TM^ nitrocellulose (0.45 μm; MSI, Westboro, MA) and blocked overnight with 6% powdered milk (BioRad) in Tris-buffered saline with Tween (TBST; Bio Rad). The membranes were probed with 1:1000 dilution of primary antibodies in TBST with 3% dry milk. The primary antibodies used were a rabbit polyclonal antibody specific for mannan (Dako, Carpinteria, CA) or a rabbit polyclonal antibody specific for *C*. *albicans* (Thermo Fisher). Primary antibodies were detected with a 1:5000 dilution of secondary goat anti-rabbit antibody conjugated with peroxidase (Sigma) in 20 mL TBST with 2% dry milk. Bound antibody was detected using a chemiluminescence kit (ECL Plus western blotting reagent, Thermo Fisher). Blotted gels were visualized using a FluorChem E imaging system (ProteinSimple, Santa Clara, CA) and gels were photograph with a Nikon X7500 camera using light-box back lighting.

### Cells and cell culture

HEK-Blue^TM^ Null 2 and HEK-Blue^TM^ hTLR4 cells (herein referred to as HEK-Null and HEK-TLR4, respectively) were purchased from InvivoGen, San Diego, CA. HEK-Null cells are stably transfected with a plasmid containing the secreted embryonic alkaline phosphatase (SEAP) reporter gene under the control of the IL-12p40 minimal promoter fused to five NF-κB binding sites. HEK-Null cells were stably transfected with human *TLR4*, *MD2*, and *CD14* genes to produce HEK-TLR4 cells. Neither cell line is known to express TLR2 or MR. The HEK-Null and HEK-TLR4 cell lines were cultured at 37°C/5% CO_2_ in DMEM supplemented with 4.5 g glucose/L (Thermo Fisher), 50 U/mL penicillin, 50 μg/mL streptomycin (Thermo Fisher), 100 μg/mL normocin (Thermo Fisher), 2 mM L-glutamine (Thermo Fisher), and 10% heat-inactivated FBS (Atlanta Biologicals, Flowery Branch, GA). HEK-Null and HEK-TLR4 cells were maintained under selection using 100 μg/mL zeocin (InvivoGen) or HEK-Blue^TM^ (InvivoGen), respectively.

### Cell stimulation and QuantiBlue assay

HEK reporter cells were grown to 50–70% confluence (viz., greater than 70% confluence results in high NF-κB background values). Cells were dislodged from adherence in cold Ca- and Mg-free Hank’s Balanced Salt Solution (HBSS). Dislodged cells were re-suspended to a density of 0.5–1×10^5^ reporter cells per ml. Each well in a 96-well flat-bottomed tissue culture plate received 100 μl of the cell suspension prior to treatment. Cells were treated immediately after plating with complete media alone or complete media plus dilutions of LPS (from *Salmonella typhimurium*; Sigma), purified CMP [14], indomethacin (Sigma), celecoxib (Sigma) or a combination of these agonists and inhibitors in a final volume of 200 μL/well. Cytotoxicity assays were performed with dilutions of celecoxib and compared with non-treated (negative control) or Triton X-100 treated reporter cells (positive cytotoxic control) [43] using an XTT (sodium 3’-[1-(phenylaminocarbonyl)-3,4-tetrazolium]-bis(4-methoxy-6-nitro) benzene sulfonic acid hydrate metabolic assay [62]. All ligand and reagent dilutions were made in complete media to prevent dilution of growth factors. Polymyxin B columns (Sigma) were used according to manufacturer’s instructions to eliminate LPS contamination from the CMP preparation prior to stimulation of HEK-TLR4 cells. A range of LPS concentrations were similarly depyrogenated with polymyxin B columns to confirm resin efficiency with our media.

NF-κB activity was determined by measuring the SEAP activity derived from NF-κB responsive SEAP reporter genes found on plasmids in the reporter cell. As per manufacturer’s specs for TLR4-reporter cells (Invivogen), SEAP accumulated in the culture medium following overnight incubations with TLR4 agonists. For each well, 20 μL of supernatant were removed and transferred to a new 96 well plate containing 180 μL pre-warmed (37°C), filtered (0.22 μm) QUANTI-Blue^TM^ (a proprietary Invivogen chromogenic substrate for SEAP). Reactions were developed at 37°C (without CO_2_) for 30–60 min and SEAP activity was measured at 630 nm using a BioTek Epoch microplate spectrophotometer. The results were evaluated with Gen5 Data Analysis software (BioTek US, Winooski, VT). HEK-Null cells served as an important control for the assay as they do not respond to TLR4 ligands, but SEAP production can be readily induced by treatment with 100 ng/mL TNFα. All tests and controls were done in triplicate and the mean SEAP activation +/- SEM were reported. Experiments were performed three times unless otherwise indicated.

### RNA isolation and cDNA synthesis

Cells were grown in 1–2 mL of complete medium to 50–70% confluence in 24-well plates (1–2 × 10^6^ cells/mL) and were treated as described in the previous section. After the indicated treatment times, total RNA was isolated using the TRIzol method (Invitrogen, Carlsbad, CA) according to the manufacturer’s instructions. RNA concentrations were measured using an ND-1000 spectrophotometer (NanoDrop Technologies, Wilmington, DE) at 260 nm. Contaminating genomic DNA was removed by treating 1 μg of each RNA preparation with 1 Unit RQ1 RNase-free DNase (Promega, Madison, WI). After 30 min, DNase was inactivated and cDNAs were synthesized using Promega’s ImProm-II™ Reverse Transcription system according to the manufacturer’s instructions. All cDNAs were analyzed by quantitative (qPCR).

### Quantitative PCR

All primers for qPCR were custom-ordered from ThermoFisher Scientific (Pittsburgh, PA). Whenever possible, primers were designed to span introns to eliminate background signals from genomic DNA. The following primers were used for qPCR: TNFα Fwd 5’-GGGACCTCTCTCTAATCAGCCCTCTGG-3’ and Rev 5’-GACGGCGATGCGGCTGATGG-3’ (290 bp, 60°C annealing; [63]), IL-8 Fwd 5’-CAAGAGCCAGGAAGAAACCA-3’ and Rev 5’-GTCCACTCTCAATCACTCTCAG -3’ (225 bp, 55°C annealing; [64]), COX-2 Fwd 5’-GAATCATTCACCAGGCAAATTG-3’and Rev 5’-TCTGTACTGCGGGTGGAACA-3’ (321 bp, 55°C annealing; [65]), GAPDH Fwd 5’-GGTATCGTGGAAGGACTCATGAC-3’ and Rev 5’-ATGCCAGTGAGCTTCCCGTTCAGC-3’ (188 bp, 55°C annealing, [66]) and β-actin Fwd 5’-ATTGCCGACGGATGCAGAA-3’ and Rev 5’-GTCATACTCCTGCTTGCTGACC-3’ (165 bp, 55°C annealing, [67]). Each PCR mixture (25 μL) was comprised of 1X iQ SYBR mix (Bio-Rad, Hercules, CA), 0.5 μM each for the forward and reverse primers, and 2 μL of cDNA or sterile H_2_O for negative controls. Reactions were run in an MJ Mini Opticon (Bio-Rad) thermal cycler using the following conditions: denaturation at 95°C for 5 min followed by 40 cycles of amplification (95°C/15 sec, 55–60°C/30 sec, 72°C/30 sec). All final PCR reactions were subjected to melt curve analyses. Data were analyzed using Bio-Rad’s CFX Manager software. Threshold values were automatically established by the software programming, and the cycle in which each sample crossed the threshold (C_T_) was recorded. The ΔC_T_ value was determined for each sample as the C_T_ value for the gene of interest–the C_T_ value for the housekeeping gene. Both GAPDH and β-actin were used as housekeeping genes. Expression in each treatment group was converted to fold expression (increase or decrease) over the average basal expression level seen in cells treated with media (set to a value of 1). All analyses were performed on triplicate samples (at a minimum) and all experiments were repeated using new cells at least 1–2 times for a total of 2–3 replications.

### Statistical analyses

Data from multiple samples are represented as the mean ± SEM (indicated by error bars). For most figures, the variance is low enough that the error bars are not visible outside of the data symbol margins. Where serial dilution or time course curves were calculated, linear regression r^2^ analyses were performed to determine the significance differences occurring between the curves. Moreover, *t*-tests were used to determine significant differences between the means. Values of significance are indicated as p ≤ 0.05 (*), p ≤ 0.005 (**), or p ≤ 0.0005 (***).
